# Supplementary figures and images for: REV-ERB agonism improves liver pathology in a mouse model of NASH
Source: PLoS One. 2020 Oct 1;15(10):e0236000. doi: 10.1371/journal.pone.0236000 (PMC7529425; doi:10.1371/journal.pone.0236000)

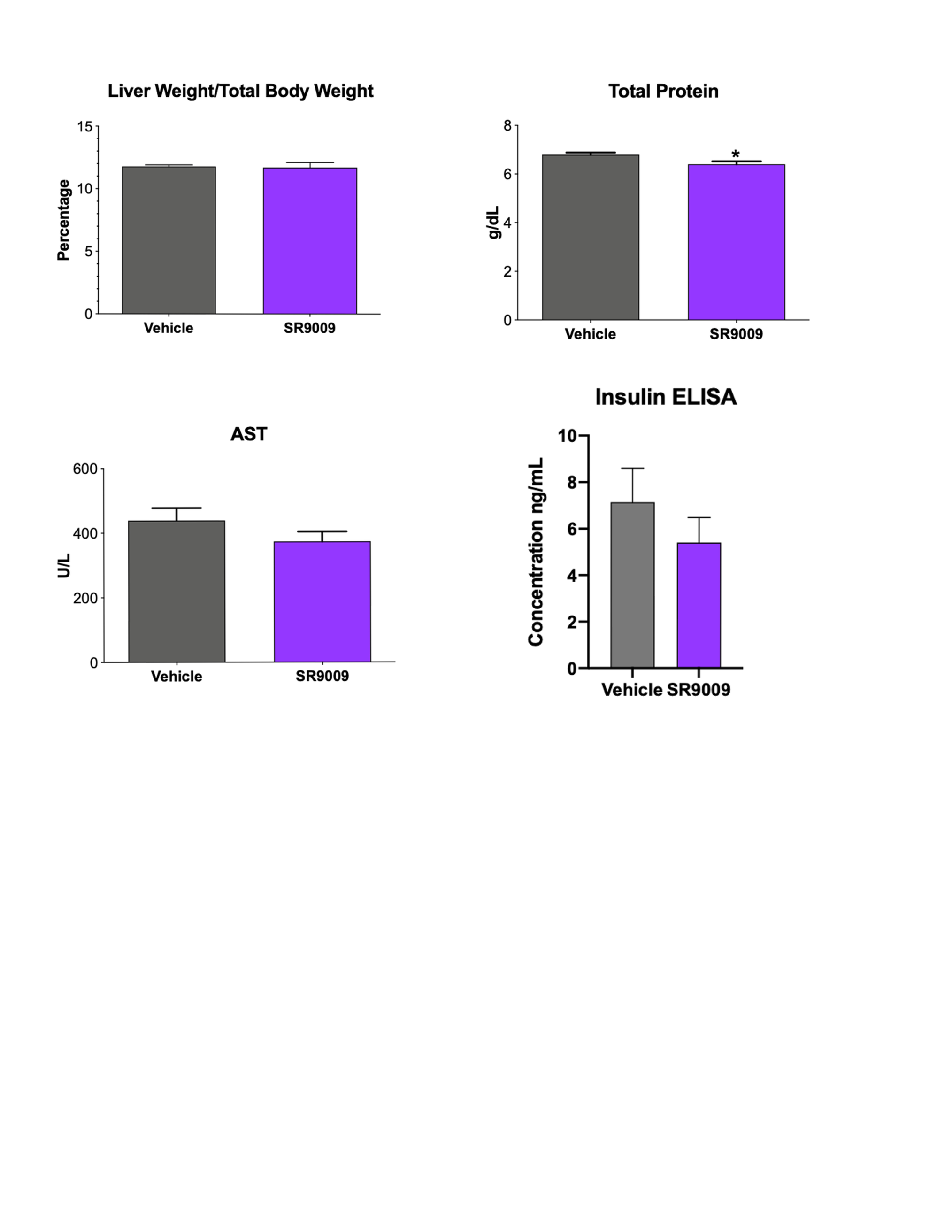

Supplement: S1 Fig — Percentage of Liver weight to total body weight, AST levels, and plasma insulin levels were not significantly changed by SR9009 treatment although total protein was significantly reduced in these mice. (TIF) [file pone.0236000.s002.tif]

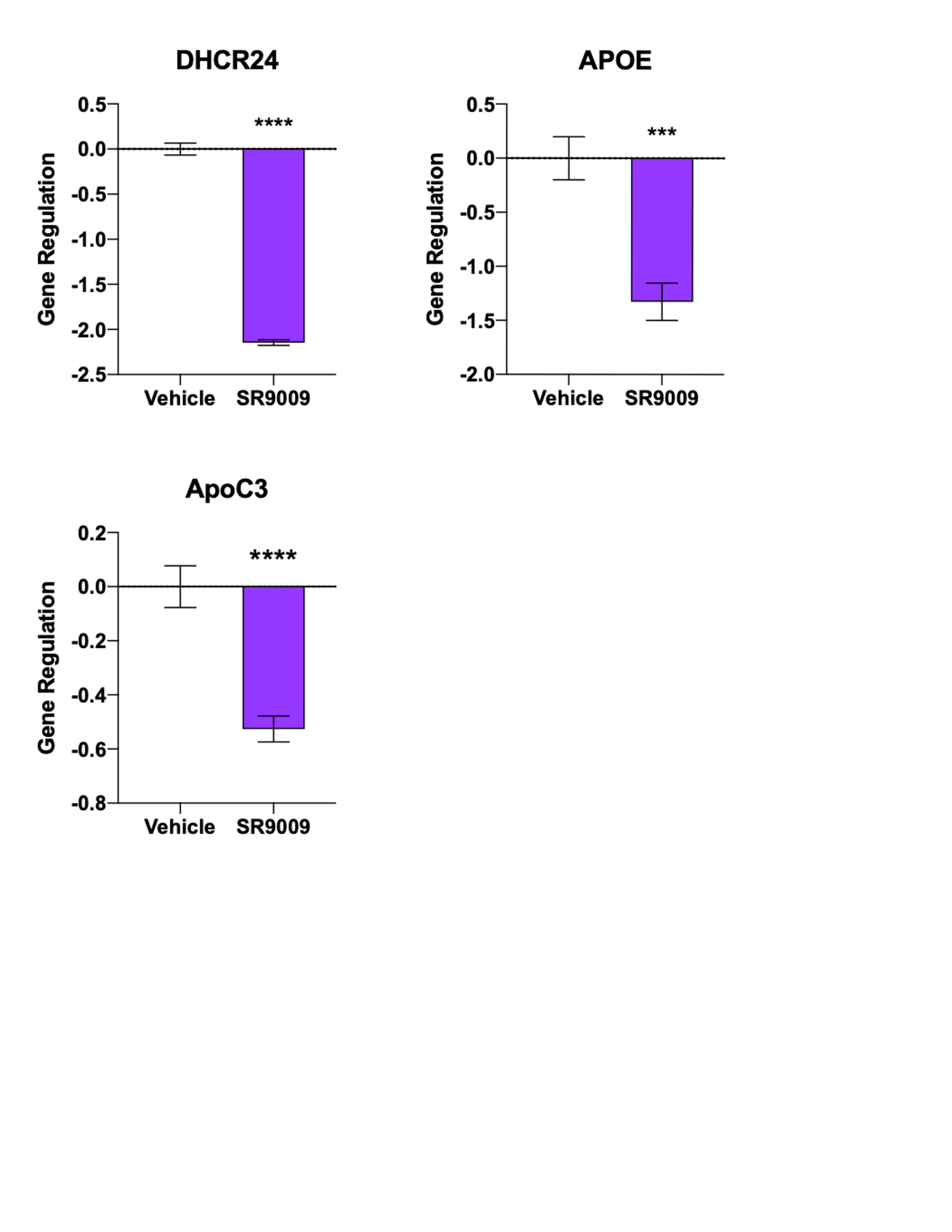

Supplement: S2 Fig — We analyzed several REV-ERB target genes (Dhcr24, ApoeE, and ApoC3) by QPCR to determine expression level differences in the groups. As indicated by the graphs, all three target genes were significantly downregulated, suggesting that SR9009 treatment was suppressing these metabolic pathways but had poor efficacy for reducing steatosis. (TIF) [file pone.0236000.s003.tif]

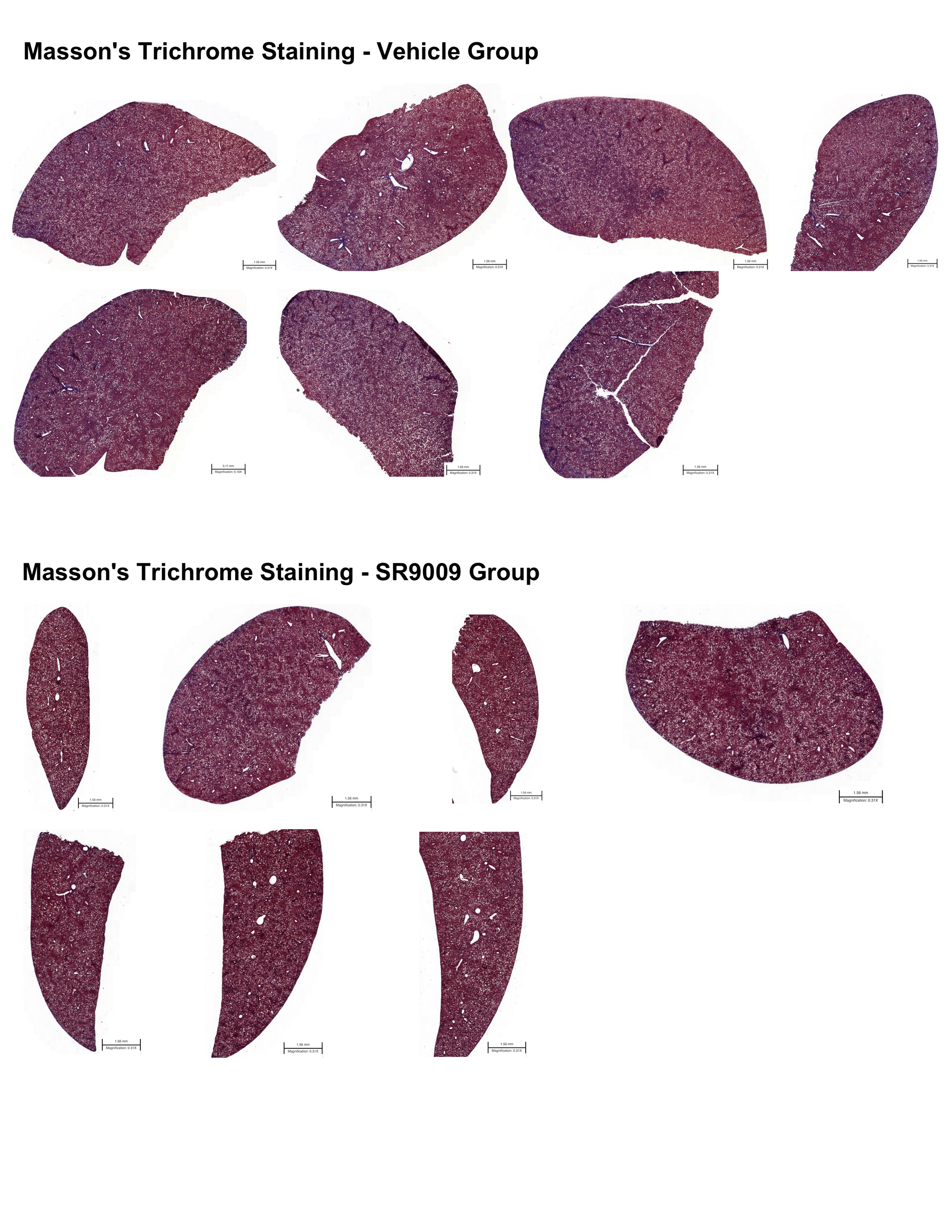

Supplement: S3 Fig — Scale bars indicate 1.56 mm. (TIF) [file pone.0236000.s004.tif]
