# Supplementary material for: REV-ERB agonism improves liver pathology in a mouse model of NASH
Source: PLoS One. 2020 Oct 1;15(10):e0236000. doi: 10.1371/journal.pone.0236000 (PMC7529425; doi:10.1371/journal.pone.0236000)
Supplement: S1 Table — (DOCX) [file pone.0236000.s005.docx]

| **Supplemental Table 1: Summary of Histo-Pathological Analysis on Liver Sections.** | | | | | | |
| --- | --- | --- | --- | --- | --- | --- |
|  |  |  |  |  |  | |
|  |  | **Vehicle-Treated** | | **SR9009-Treated** | | |
| **Steatosis** | | Mean | SEM | Mean | SEM | |
|  | % Lipid Area | 10.6699 | 0.5175 | 11.1016 | 0.7272 | |
|  | Total Lipid Area (mm^2^) | 23.76 | 1.3301 | 21.78 | 2.04 | |
|  | % Macrovesicular | 92.22 | 0.4001 | 92.43 | 0.3693 | |
|  | % Microvesicular | 7.776 | 0.4004 | 7.664 | 0.3693 | |
|  | Average Vesicle Size (μm) | 150.9 | 6.649 | 150.9 | 6.573 | |
|  |  |  |  |  |  | |
| **Ballooning and Mallory Bodies** | |  |  |  |  | |
|  | Ballooning Hepatocyte Density (Cells/mm^2^) | 78.38 | 5.6537 | 66.84 | 6.3194 | |
|  | Mallory Bodies Present | Present | | Present | | |
|  |  |  |  |  |  | |
| **Inflammation** | |  |  |  |  | |
|  | Immune Cell Density (Cells/mm^2^) | 841.092 | 74.4559 | 755.943 | 28.3342 | |
|  | Immune Cell Count | 186998 | 16648 | 151180 | 16227 | |
|  | Total Immune Cell Area (mm^2^) | 20.59 | 1.0573 | 19.05 | 1.9442 | |
|  |  |  |  |  |  | |
| **Fibrosis** | |  |  |  |  | |
|  | % Fibrosis Area | 3.479 | 0.3304 | 1.039 | 0.2401 | |
|  | Total Fibrosis Area (mm^2^) | 7.792 | 0.8168 | 1.9015 | 0.37101 | |
|  | | | | | | |
